# Structural determinants of an internal ribosome entry site that direct translational reading frame selection

**DOI:** 10.1093/nar/gku622

**Published:** 2014-07-18

**Authors:** Qian Ren, Hilda H.T. Au, Qing S. Wang, Seonghoon Lee, Eric Jan

**Affiliations:** Department of Biochemistry and Molecular Biology, University of British Columbia, Vancouver, BC V6T 1Z3, Canada

## Abstract

The dicistrovirus intergenic internal ribosome entry site (IGR IRES) directly recruits the ribosome and initiates translation using a non-AUG codon. A subset of IGR IRESs initiates translation in either of two overlapping open reading frames (ORFs), resulting in expression of the 0 frame viral structural polyprotein and an overlapping +1 frame ORFx. A U–G base pair adjacent to the anticodon-like pseudoknot of the IRES directs +1 frame translation. Here, we show that the U-G base pair is not absolutely required for +1 frame translation. Extensive mutagenesis demonstrates that 0 and +1 frame translation can be uncoupled. Ribonucleic acid (RNA) structural probing analyses reveal that the mutant IRESs adopt distinct conformations. Toeprinting analysis suggests that the reading frame is selected at a step downstream of ribosome assembly. We propose a model whereby the IRES adopts conformations to occlude the 0 frame aminoacyl-tRNA thereby allowing delivery of the +1 frame aminoacyl-tRNA to the A site to initiate translation of ORFx. This study provides a new paradigm for programmed recoding mechanisms that increase the coding capacity of a viral genome.

## INTRODUCTION

Protein synthesis is a highly accurate process where errors in translation occur at low frequency about 5 × 10^−5^ per codon. Although we have a basic understanding of transfer ribonucleic acid (tRNA) discrimination by the ribosome, the maintenance of the open reading frame (ORF) during translation is still not well understood. Viral strategies have been particularly informative, as compact viral genomes have evolved mechanisms to increase their coding capacities for successful infection. In particular, programmed frameshifting can increase coding capacity in viral genomes and in some cellular messenger RNAs (mRNAs) (1–3).

We recently discovered a novel recoding mechanism found within a subset of the *Dicistroviridae* family (4). Dicistroviruses possess a positive-sense, single-stranded RNA (ssRNA) genome that contains two main ORFs encoding the non-structural and structural viral proteins, respectively (5,6). Different internal ribosome entry sites (IRESs) direct translation of the two main ORFs (7). The intergenic (IGR) IRES has several unique properties; the IRES can recruit the ribosome without the need of initiation factors and initiates translation from a non-AUG codon in the ribosomal A site (8). The IGR IRES adopts a structure containing three pseudoknots (PKI–III). PKII/PKIII form a compact structure that is responsible for ribosome recruitment and binding (9–12). Structural and biochemical studies have revealed that the PKI domain adopts a tRNA anticodon–codon-like interaction that occupies the ribosomal P site to start translation by delivery of the first aminoacyl-tRNA to the A site (13,14). A recent cryo-electron microscopy (EM) structure of the CrPV IGR IRES bound to the yeast ribosome at 3.7–3.8-Å resolution has provided additional insights into the IGR IRES mechanism: the PKI domain first occupies the ribosomal A site and translocation of the IRES by eEF2 occurs prior to delivery of the first aminoacyl-tRNA (15). Subsequently, the PKI domain occupies the ribosomal P site to drive translation from the ribosomal A site using a non-AUG codon. Thus, the IRES hijacks and manipulates the ribosome by functionally mimicking a tRNA. After ribosome recruitment, eEF1A mediates delivery of the first aminoacyl-tRNA to the A site and eEF2 catalyzes the initial translocation step in the absence of peptide bond formation, termed pseudotranslocation (8,16,17).

**Figure 1.**
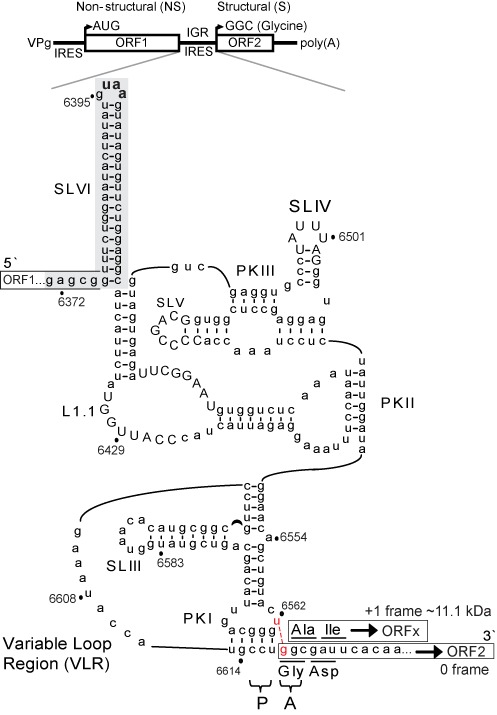
Secondary structure of the IAPV IGR IRES. (Top) Schematic organization of IAPV genome. Distinct IRES direct translation of nonstructural (ORF1) and structural (ORF2) polyproteins. (Bottom) Schematic of IAPV IGR IRES showing pseudoknots PKI, PKII and PKIII, stem loops SLIII, SLIV, SLV and SLVI (shaded gray) and loop L1.1. The UAA stop codon of ORF1 is shown in bold within the loop of SLVI. The overlapping +1 frame ORFx, within the 0 frame ORF2, is shown. Conserved nucleotides among type II IGR IRESs are in capital letters. The CCU triplet, in part, mediates PKI base pairing and occupies the P site, whereas the first codon of the 0 frame ORF2 is the adjacent GGC codon in the A site. Translation of the +1 frame ORFx is directed by a U6562/G6618 base pair adjacent to PKI (in red nucleotides). The start codon in the +1 frame is a GCG alanine codon.

Bioinformatics studies have revealed a hidden gene called ORFx which is downstream of the IRES and overlaps with the structural protein ORF within a subset of dicistrovirus genomes including the fire ant virus, *Solenopsis invicta virus*-1 (SINV-1) and the honey bee viruses, Israeli acute paralysis virus (IAPV), Acute bee paralysis virus (ABPV) and Kashmir bee virus (KBV) (18,19). We previously showed that the IAPV IGR IRES directs translation of ORFx in the +1 frame that is dependent on a U6562/G6618 base pair adjacent to the PKI tRNA-like domain (Figure 1) (4). Moreover, an ORFx peptide was detected in virally infected honey bees by mass spectrometry analysis, suggesting a role of ORFx during virus infection (4). This work demonstrated a novel recoding mechanism utilizing an IRES to initiate translation in the 0 and +1 frames to produce two overlapping gene products and suggested a ‘yardstick’ model in which the adjacent U–G base pair shifts the reading frame by +1. In this study, we expand on this model and provide extensive analyses using mutagenesis and SHAPE to show that the IRES adopts distinct conformations to direct reading frame selection. In addition to the U–G adjacent base pair, we have uncovered novel classes of mutations that direct only 0 or +1 frame translation. Finally, ribosome positioning experiments suggest that the viral IRES directs reading frame selection after ribosomes have assembled on the IRES. This work describes a novel strategy to alter the ribosome reading frame to increase the coding capacity of a viral genome.

## MATERIALS AND METHODS

### Plasmids

The bicistronic and the monocistronic luciferase plasmids containing the IGR IRES of IAPV (NC_009025) (nucleotides 6399–6908), KBV (NC_004807) (nucleotides 6381–6908), ABPV (NC_002548) (nucleotides 6296–6814) and SINV-1 (NC_006559) (nucleotides 4189–4797) have been described (4). The IAPV IGR IRES and the downstream region (NC_009025, nucleotides 6372–6908) were subcloned within the intercistronic region between the reporter luciferase genes of the bicistronic constructs. Inclusion of the surrounding sequences facilitates IRES translation (4). Mutants were generated by polymerase chain reaction-based site-directed mutagenesis and confirmed by sequencing.

### *In vitro* translation assays

Bicistronic luciferase plasmids, linearized with XbaI, were incubated in Sf21 extract (Promega) with an additional 40-mM potassium acetate and 0.5-mM magnesium chloride (final concentration) for 2 h at 30°C in the presence of [^35^S]-methionine (PerkinElmer). Reactions were analyzed by sodium dodecyl sulphate-polyacrylamide gel electrophoresis (SDS-PAGE) and by phosphorimager analysis (Typhoon, Amersham).

Monocistronic luciferase-containing plasmids were linearized with SpeI. IAPV IGR IRES RNAs were transcribed *in vitro* using a bacteriophage T7 RNA polymerase reaction and purified with an RNeasy kit (Qiagen). The integrity and purity of the transcribed RNAs were confirmed by gel analysis.

### *In vivo* translation assay

We previously demonstrated that the fusion of ORFx in-frame with the Firefly luciferase (FLuc) ORF inhibits FLuc enzymatic activity and the inclusion of the *Thosea asigna* virus 2A peptide (T2A) into the bicistronic reporter system increases the sensitivity of the luciferase assay (20). Capped reporter RNAs from bicistronic T2A containing plasmids were generated by *in vitro* transcription in the presence of a cap analog [m^7^G(5′)ppp(5′)G] (Ambion) at a 5:1 ratio to GTP. *Drosophila* S2 cells were grown and passaged in M3+BPYE medium plus 10% fetal bovine serum (FBS) at 25ºC. Capped bicistronic reporter RNAs (2 μg) were transfected into 3 × 10^6^ S2 cells with Lipofectamine 2000 (Invitrogen). After 6 h of transfection, cells were harvested, lysed and luciferase activity was measured by dual-luciferase reporter assay (Promega) as previously described (20).

### SHAPE probing

SHAPE probing was performed as described (21). An amount of 20 pmol of the RNA was heated to 85°C for 2 min, followed by the addition of Buffer E (final concentration of 20-mM Tris, pH 7.5, 0.1-M KCl, pH 7.0, 2.5-mM MgOAc, 0.25-mM spermidine and 2-mM dithiothreitol (DTT) and incubated at 30°C for 20 min. To modify the RNA, *N*-methylisatoic anhydride (NMIA) dissolved in dimethyl sulfoxide (DMSO) was added (final concentration 3 mM) and incubated for 1.5 h at 30°C. Control reactions containing only DMSO (no NMIA) were performed in parallel. The modified RNA was recovered by ethanol precipitation, followed by primer extension analysis. Reactions were resolved on 8% (w/v) denaturing polyacrylamide sequencing gels at 60 W. The gels were dried and analyzed by phosphorimager analysis. Individual band intensities were quantitated using semi-automated footprinting analysis (SAFA) (22) and normalized as described (23). Band intensity variations for each nucleotide were normalized by dividing all intensities by the average value of the next 10% of reactive nucleotides, after excluding the top 2% of the reactive nucleotides (23).

For SHAPE analysis of IRES/ribosome complexes, 20 pmol of the RNA was heated to 85°C for 2 min, followed by the addition of Buffer E (final concentration of 20-mM Tris, pH 7.5, 0.1-M KCl, pH 7.0, 2.5-mM MgOAc, 0.25-mM spermidine and 2-mM DTT) and incubated at 30°C for 20 min. 80S/IRES complexes were formed by adding 20 pmol of purified 80S Hela ribosomes to the folded RNA for 10 min at 30°C. To modify the RNA, NMIA was added to the reaction at a final concentration of 3 mM and incubated for 1.5 h at 30°C. IRES–ribosome complexes were separated by ultracentrifugation through 10–35% (w/v) sucrose gradients containing 20-mM Tris, pH7.5, 0.1-M KCl, pH 7.0, 5-mM MgOAc, 0.25-mM spermidine, 2-mM DTT. After fractionation, IRES–80S complexes were collected, ethanol-precipitated and resuspended in water. Primer extension was performed and reactions were resolved on 8% (w/v) denaturing polyacrylamide sequencing gels at 60 W. The gels were dried and analyzed by phosphorimager analysis. Individual band intensities were quantitated using SAFA and normalized as described (22). Control experiments including reactions in the absence of NMIA or ribosomes were performed in parallel. The difference in SHAPE reactivities of nucleotides between wild-type and mutant IRES/ribosome complexes was calculated. The averages and standard deviations were calculated from at least three independent experiments.

### Purification of 40S and 60S ribosomal subunits

Ribosomal subunits were purified from HeLa cell pellets (National Cell Culture Center). HeLa cells were lysed in Triton-X lysis buffer (15-mM Tris-HCl (pH 7.5), 300-mM NaCl, 1% (v/v) Triton X-100, 6-mM MgCl_2_, 1-mg/ml heparin) and the supernatant was subjected to brief centrifugation to remove cellular debris. The supernatant was applied to a 30% (w/w) sucrose cushion containing 0.5-M KCl and centrifuged at 100,000 g to pellet ribosomes. Ribosomes were resuspended in buffer B (20-mM Tris-HCl (pH 7.5), 6-mM magnesium acetate, 150-M KCl, 6.8% (w/w) sucrose, 2-mM DTT) and subsequently treated with puromycin to dissociate ribosomes from the mRNAs. KCl was added to obtain a final concentration of 0.5 M. The dissociated ribosomes were resolved on a 10–30% (w/w) sucrose gradient where the peaks corresponding to the free 40S and 60S subunits were detected by measuring the absorbance at 260 nm. The corresponding fractions were collected, pooled and concentrated in buffer C (20-mM Tris-HCl (pH 7.5), 0.2-mM ethylenediaminetetraacetic acid (EDTA), 10-mM KCl, 1-mM MgCl2, 6.8% (w/w) sucrose) using Amicon Ultra spin concentrators (Millipore). The resultant concentrations of the ribosomal subunits were determined by spectrophotometry using the conversions 1 A260 nm = 50 nM and 1 A260 nm = 25 nM for the 40S and 60S subunits, respectively.

### Toeprinting analysis

Toeprinting analysis of ribosomal complexes was performed as previously described (9). An amount of 150 ng of bicistronic wild-type or mutant IGR IRES RNAs were annealed to primer PrEJ761 (5′-CATGGGGGTATCGATCCTATTTGGAG-3′) in 40-mM Tris (pH 7.5) and 0.2-mM EDTA by slow cooling from 65°C to 37°C. Following primer annealing, the RNAs were incubated with 100-nM or 150-nM purified HeLa 40S and 60S, respectively. Ribosome positioning was determined by primer extension/reverse transcription using five units of Avian Myeloblastosis Virus (AMV) reverse transcriptase (Promega), 415 μM of each of deoxythymidine triphosphate (dTTP), deoxyguanosine triphosphate (dGTP) and deoxycytidine triphosphate (dCTP), and 83-μM of deoxyadenosine triphosphate (dATP), 0.33 μl of α-[^32^P] dATP (3.33 μM, 3000 Ci/mmol) and 6.7-mM MgOAc in the final reaction volume. Following incubation at 37°C for 1 h, the samples were extracted by phenol/chloroform (twice), chloroform alone (once) and ethanol-precipitated. The complementary deoxyribonucleic acids (cDNAs) were analyzed under denaturing conditions on 6% (w/v) polyacrylamide/8-M urea gels, which were subsequently dried and subjected to phosphorimager analysis.

### Reconstitution of IRES–ribosome translation

To reconstitute translocation on IRES–ribosome complexes, 150-ng bicistronic wild-type or mutant RNAs were annealed to primer PrEJ761 as described above. Following primer annealing, the RNAs were incubated with 100-nM or 150-nM purified HeLa 40S and 60S subunits, respectively, in the presence of 1-mM ATP, 0.4-mM GTP and 0.5-mg/ml cycloheximide. Purified yeast elongation factor 1A (30 ng/μl) and elongation factor 2 (50 ng/μl) and bulk aminoacyl-tRNAs were added to promote translocation. Reverse transcription and visualization of cDNA products were performed as above.

## RESULTS

### Base pairing between nucleotides 6562 and 6618 is important but not absolutely necessary for +1 frame translation

**Figure 2.**
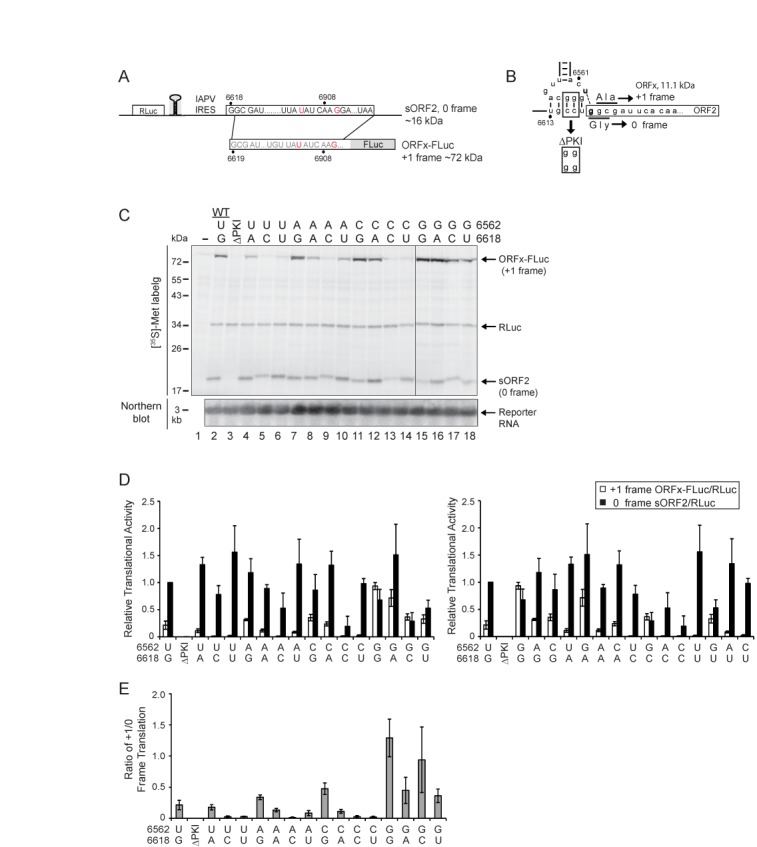
Characterization of the U6562/G6618 base pair adjacent to the PKI domain. (**A**) Schematic of bicistronic reporter constructs containing the wild-type or mutant IAPV IGR IRES inserted in the intergenic region between two reporter genes: Renilla luciferase (RLuc), which monitors scanning-mediated translation, and firefly luciferase (FLuc), which monitors IRES-mediated translation. Mutations within ORFx (in red nucleotides) were engineered to fuse ORFx in frame with FLuc to produce ORFx-FLuc. The mutations also result in a truncated 0 frame ORF2 protein, sORF2. (**B**) PKI domain of the IAPV IGR IRES. The CC6615-6GG mutation that disrupts PKI base pairing is shown. The U6562 and G6618 are in bold. (**C**) The effects of mutating U6562 and G6618 on IAPV IGR IRES-mediated 0 and +1 frame translation. Bicistronic reporter constructs were incubated in Sf21 extracts at 30°C for 120 min in the presence of [^35^S]-methionine. In parallel, RNA extracted from reactions were probed by northern blot analysis (below). (**D**) Quantitation of the radiolabeled +1 frame ORFx-Fluc (white bars) and 0 frame sORF2 (black bars) proteins normalized to RLuc. The ratios are normalized to the 0 frame translational activity by the wild-type IRES. The data are plotted by either grouping nucleotide changes to U6562 (left graph) or G6618 (right graph). (**E**) Data plotted as a ratio of +1 to 0 frame translation. Averages are shown from at least three independent experiments ± SD.

Previously, we demonstrated that the U6562/G6618 base pair adjacent to the IAPV PKI domain (see Figure 1) directs +1 frame translation of ORFx (4). To examine in detail whether the nucleotide identities of U6562 and G6618 are important for 0 or +1 frame translation, we generated mutant IRESs containing all 16 base permutations at positions 6562 and 6618 (Figure 2B–E). IRES-mediated 0 and +1 frame translation were assessed by using a bicistronic reporter construct, in which the downstream +1 frame ORFx is fused in-frame with FLuc to produce an ORFx-FLuc fusion protein (∼72 kDa) (Figure 2A). This fusion also results in a shortened 0 frame ORF2 (Figure 2A, sORF2). Thus, scanning-dependent Renilla luciferase (RLuc) and IGR IRES-mediated 0 and +1 frame translation are monitored simultaneously from the same construct. The translational activities were analyzed by incubating the reporter constructs in an insect Sf21 coupled transcription/translation extract containing [^35^S]-methionine, followed by quantifying the radiolabeled protein products by SDS-PAGE analysis. As shown previously, Northern blot analyses showed that the bicistronic RNAs synthesized in these extracts are intact and full-length (Figure 2C) (4). The translational activities of the 16 different permutations are summarized in Figure 2D and Supplementary Figure S1 in two graphs that are organized based on the base identities at positions 6562 and 6618. Figure 2D shows the relative translational ratio of +1 frame to 0 frame, whereas Supplementary Figure S1 normalizes the translational activities to both the wild-type 0 and +1 frame translation set as 1. Depending on the identity of the bases at positions 6562 and 6618, the efficiency of translation in either 0 or +1 frame as well as the ratio of efficiencies varied considerably. G6618 mutated to C or U but not to A abolished +1 frame translation, consistent with the model that base pairing between nucleotides 6562 and 6618 is necessary for +1 frame translation (Figure 2C, lanes 2, 4–6; Figure 2D) (4). In support of this model, combinations that maintained base pairing such as U6562A/G6618U, U6562C/G6618, U6562G/G6618C and U6562G/G6618U (Figure 2C, lanes 10, 11, 17, 18) rescued +1 frame translation albeit to different extents (4), whereas mutation combinations that do not base pair, such as U6562A/G6618C, U6562C/G6618C, and U6562/G6618U, inhibited +1 frame translation, thus reinforcing the importance of base pairing between nucleotides 6562 and 6618. However, surprisingly, Watson–Crick base pairing is not a strict requirement for mutant IRESs with position 6618 as G or A. For example, mutation of U6562 to G or A (Figure 2C, lanes 7 and 15) or a combination of U6562G/G6618A (Figure 2C, lane 16), all of which should not form a Watson–Crick 6562/6618 base pair, resulted in a significant level of +1 frame translation. Interestingly, specifically mutating U6562 to G regardless of the nucleotide identity of 6618 significantly increased +1 frame translation (Figure 2D, left graph, and Supplementary Figure S1B). This is most evident when the ratio of +1/0 frame translation is plotted (Figure 2E). The mutant IRES U6562G, which increased +1 frame translation by ∼4.5-fold as compared to the wild-type IRES, displayed similar levels of 0 frame translation (Figure 2D and Supplementary Figure S1). Altering the first +1 frame GCG codon to a stop UAG codon in combination with the U6562G mutation eliminated +1 frame translation, confirming that translation starts at the +1 frame GCG codon (Qian,R., unpublished data). As shown previously, mutating CC6615-6 to GG which disrupts PKI base pairing eliminated both 0 and +1 frame translation (Figure 2B; Figure 2C, lane 3), whereas compensatory mutations that restore base pairing rescued translation in both frames (Supplementary Figure S1B and C), demonstrating that 0 and +1 frame translation are dependent on the integrity of PKI and are IGR IRES-dependent (4). In summary, these results demonstrate that +1 frame translation mediated by the wild-type IRES (for simplicity, we will refer to the IAPV IGR IRES as IRES unless specifically stated) is dependent on base pairing between nucleotides 6562 and 6618. However, in specific mutant IRES contexts, the adjacent base pairing is not absolutely necessary for +1 frame translation.

**Figure 3.**
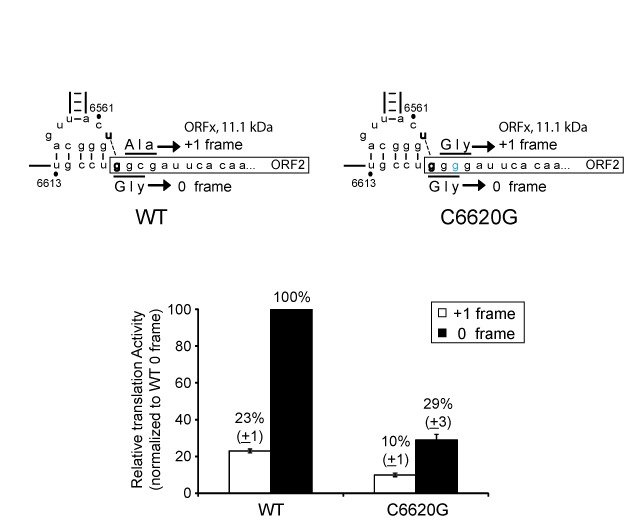
Reading frame selection directed by the mutant C6620G IRES. (Top) Schematic of wild-type and C6620G mutant IRESs are shown. Mutating C6620 to G results in an initiating GGG start codon in both the 0 and +1 frames. (Bottom) IRES-mediated 0 and +1 frame translation are plotted and normalized to the wild-type IRES-mediated 0 frame translation given as 100%. Averages are shown from at least three independent experiments ± SD.

The wild-type IAPV IRES directs +1 frame translation at ∼20–25% of 0 frame translation *in vitro* (Figure 2C–E and Supplementary Figure S1) (4). We reasoned that this may be due to differences in the levels of or in the delivery efficiency of the incoming aminoacyl-tRNA to the 0 or +1 frame initiating codon. To address the latter possibility, we mutated the +1 frame GCG alanine codon to a GGG glycine codon (C6620G), such that both the 0 and +1 initiate with the GGG glycine codon and should utilize the same Gly-tRNA for delivery to the A site (Figure 3). Compared to the wild-type IRES, the C6620G mutant IRES displayed a lower level of translation in both frames, which is likely due to the particular Gly-tRNA that is delivered to the GGG codons in the 0 and +1 frames (Figure 3). Comparing the ratio of +1/0 frame translation, +1 frame translation occurred at 30% frequency of 0 frame translation for the C6620G mutant (Figure 3, 10% versus 29%), thus reflecting the true ratio of +1 to 0 frame translation mediated by the IAPV IRES.

### SHAPE analysis of the wild-type IAPV IGR IRES

**Figure 4.**
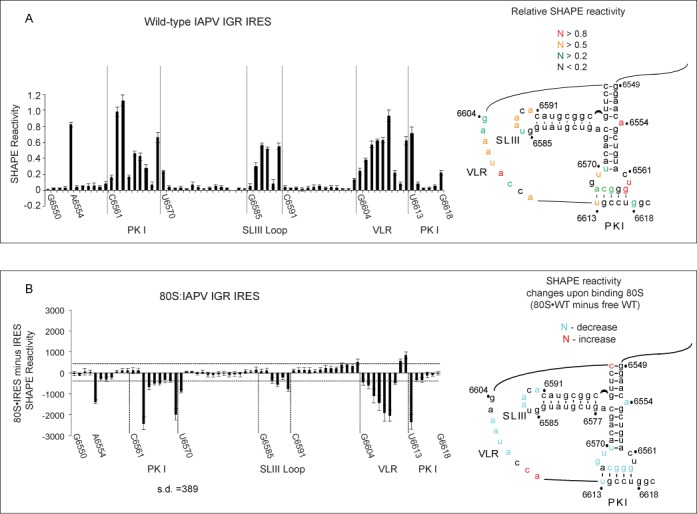
Selective 2′ hydroxyl acylation analyzed by primer extension (SHAPE) of the PKI domain of the wild-type IAPV IGR IRES. (**A**) Normalized NMIA reactivity profile of the PKI domain within the IAPV IGR IRES. The nucleotide position/number is shown on the x-axis with major domains of the IRES denoted. The y-axis is normalized reactivity. To the right, SHAPE data superimposed on the secondary structure of the IAPV IGR IRES PKI domain. The color coding indicates the relative NMIA reactivities of individual nucleotides. Nucleotides are colored in black, green, orange and red from unreactive (<0.2) and limited reactivity (0.2–0.5) to moderately (0.5–0.8) and highly reactive (>0.8). (**B**) Quantitation of the change in the NMIA reactivity of the indicated IAPV IGR IRES in IRES–ribosome complexes compared to IRES in solution. The difference in NMIA reactivity (radioactivity) of each nucleotide within the PKI domain is shown. To the right, secondary structure of the PKI domain is shown. Nucleotides that showed a significant increase (red) or decrease (blue) (greater than 1 SD) in NMIA reactivity are labeled. Data represent the average of at least three independent experiments ± SD.

Our mutagenesis analyses suggest that domains within the IRES other than base pairing between nucleotides 6562 and 6618 may contribute to +1 frame translation. By extension, we hypothesize that the IRES may adopt different conformations to direct either 0 or +1 frame translation. To address this, we probed the structure of the wild-type IAPV IRES using selective 2′-hydroxyl acylation analyzed by primer extension (SHAPE) analysis. Briefly, reactivity of nucleotides to chemicals such as NMIA reveals the flexibility of nucleotides within the RNA structure (21). Using primer extension analysis, the location and extent of modification of each nucleotide are determined by comparing band intensities from the modification reaction to those of an unmodified control. We focused our attention on the PKI domain as this region likely directs +1 frame translation. For the wild-type IRES, nucleotides that were reactive to NMIA were observed within single-stranded regions of the Variable Loop Region (VLR), the loop of SLIII, the unpaired A6554 bulge and U6562 (Figure 4A and Supplementary Figure S2). In general, nucleotides within predicted helical stems and base pairs were not reactive to NMIA (Figure 4). The one exception is within PKI. Specifically, nucleotides G6563, GCA6565-7 and U6613 within the wild-type IRES were reactive to NMIA (Figure 4A). These results are in agreement with previous reports that the PKI domain may be dynamic and is reactive toward chemicals and enzymes that recognize both ssRNA and double-stranded RNA (9,10).

We next interrogated the structure of the IRES bound to the ribosome. Previous studies have shown that the PKI domain of the related dicistrovirus, CrPV, undergoes subtle conformational changes upon ribosome binding (13). The IAPV IGR IRES was complexed with salt-washed-purified human ribosomes, subjected to NMIA, followed by sucrose gradient fractionation to isolate IRES–ribosome complexes. The IRES RNA was purified and sites of modification were analyzed by primer extension. The differences in NMIA reactivities of nucleotides of IRES alone versus IRES–ribosome complexes are shown in Figure 4B. In general, nucleotides that were NMIA-reactive within the IRES in solution such as A6554, U6613, PKI and VLR showed decreases in NMIA reactivity upon IRES binding to the ribosome. This suggests that these nucleotides become less flexible upon ribosome binding. In contrast, C6603 and CA6611-2 were more reactive in IRES–ribosome complexes than in the isolated IRES, indicating that these nucleotides become more flexible when the ribosome is assembled on the IRES.

### Novel mutant IAPV IGR IRESs that direct 0 and +1 frame translation

**Figure 5.**
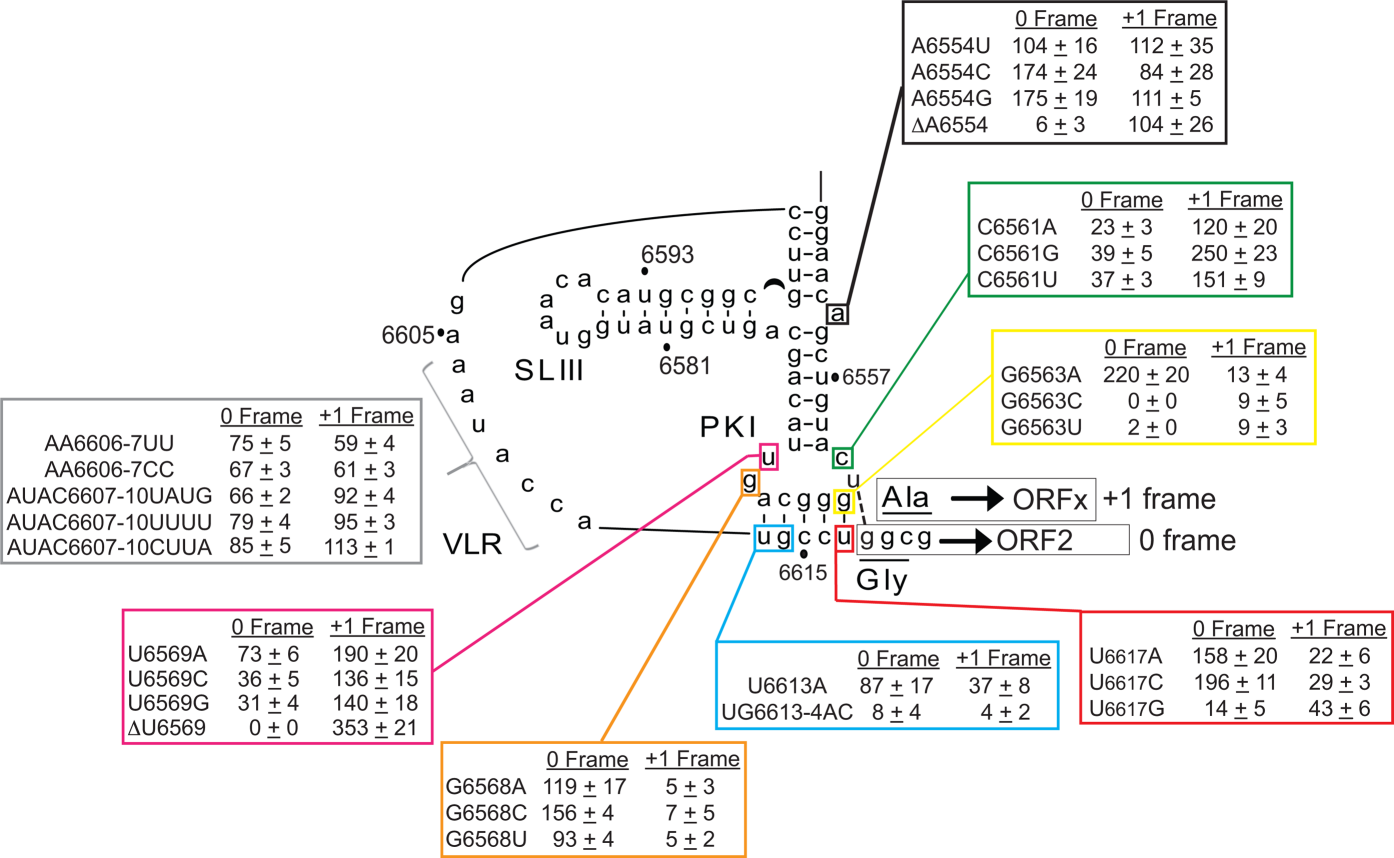
Identification of novel elements important for IAPV IGR IRES-mediated 0 and +1 frame translation. Quantitation of mutant IAPV IRES-mediated 0 and +1 frame translation in Sf21 extracts using bicistronic reporter constructs. The percent translation is normalized to the 0 and +1 frame translation (both as 100%) mediated by the wild-type IAPV IGR IRES. Shown are averages from at least three independent experiments ± SD.

The SHAPE analysis of the wild-type IAPV IRES suggests that the IRES may undergo conformational changes upon ribosome binding, especially within ssRNA regions and the PKI base pairing domain. To investigate this more closely, we systematically engineered mutations within the IRES guided by the SHAPE analysis. IRES-mediated 0 and +1 frame translation were monitored using the bicistronic reporter construct described in Figure 2A. Figure 5 summarizes the data by calculating the percentage of 0 and +1 frame translation normalized to the wild-type IRES (given as 100%).

#### PKI base pairing is required for 0 and +1 frame translation

We first focused on the anticodon-like PKI domain since the majority of changes in SHAPE reactivities occur in this region. The nucleotides on the upper strand (residues 6563–6567) within PKI were sensitive to NMIA reactivity. We reasoned that strengthening or weakening the base pairs in PKI may differentially affect 0 or +1 frame translation. We chose to mutate the bottom strand in order to not disrupt the anticodon-like loop of PKI. Mutating U6613A had a deleterious effect on +1 frame translation (∼60% decrease) but did not significantly affect 0 frame translation (Figure 5, blue box). Further disruption of PKI by changing UG6613-4 to AC, abolished both 0 and +1 frame translation, indicating that a minimum of four base pairs is required for IRES activity and translation in either frames (Figure 5, blue box). To further confirm this result, we mutated U6617, which is adjacent to the GGC start codon, to the other bases (Figure 5, red box). Mutating U6617 to G, at the most right base pair of PKI, decreased 0 and +1 frame translation by ∼85 and 60%, respectively (Figure 5, red box). In contrast, strengthening the base pair by changing U6617 to C increased 0 frame translation by 2-fold and decreased +1 frame translation by 70%. However, changing U6617 to A, which should disrupt the same base pair, increased 0 frame translation but decreased +1 frame translation (Figure 5, red box). The latter result suggests that the nucleotide identity may be important in certain contexts. In general, U6617 is absolutely necessary for optimal +1 frame translation. Moreover, strengthening the right most base pair from G6563/U6617 to G6563/U6617C increases 0 frame translation.

To complement our investigation of the right side of PKI, we introduced mutations into position G6563. Mutating G6563 to A, allowing base pairing in PKI, increased 0 frame translation and decreased +1 frame translation (Figure 5, yellow box). In contrast, mutating G6563 to C or U eliminated both 0 and +1 frame translation (Figure 5, yellow box), which is in agreement that base pairing between nucleotides 6563 and 6617 is important for IRES-mediated 0 frame translation. In summary, these results suggest that all five base pairs in PKI are required for IRES-mediated +1 frame translation. In contrast, 0 frame translation can tolerate modest disruption of PKI. Finally, our data rule out the hypothesis that +1 frame translation is due to a realignment of base pairs in PKI to shift the reading frame.

#### Mutation of unpaired nucleotides within the PKI domain

Phylogenetic analysis of the Type II IGR IRES secondary structures predicts that the PKI anticodon-like loop contains four unpaired nucleotides (Supplementary Figure S3A). Within the IRES, the unpaired nucleotides of PKI, C6561, U6562, G6568 and U6569 showed differential reactivities to NMIA by SHAPE probing (Figure 4). In particular, C6561 and G6568 were not reactive to NMIA (Figure 4A). To examine these nucleotides further, we mutated them to other bases.

Mutating G6568 to A, C or U inhibited +1 but not 0 frame translation (Figure 5, orange box). In contrast, mutating C6561 to A, U or G decreased 0 frame translation by 60–75% and increased +1 frame translation up to 2.5-fold (Figure 5, green box). These data show that the identities of C6561 and G6568 are important for the selection of 0 or +1 frame translation.

U6569 is predicted to be unpaired within the anticodon-like loop of PKI and is reactive to NMIA by SHAPE (Figure 4A). Deletion of U6569 (ΔU6569) surprisingly resulted in complete loss of 0 frame translation and a significant increase in +1 frame translation (∼3.5-fold of wild-type) (Figure 5, magenta box). Furthermore, mutating U6569 to A, C or G decreased 0 frame translation and increased +1 frame translation (Figure 5, magenta box). Thus, U6569 is important for 0 but not +1 frame translation.

A6554 is predicted to be unpaired and is highly sensitive to NMIA (Figure 4A). Mutating A6554 to U, C or G did not have an effect on +1 frame translation but increased 0 frame translation by 70% when mutated to C or G (Figure 5, black box). Interestingly, deletion of A6554 (ΔA6554) abolished 0 frame but not +1 frame translation, suggesting that the unpaired A6554 is required for 0 frame translation (Figure 5, black box). Specifically, it is noteworthy that deletion of U6569 or A6554 essentially abolished 0 frame translation and maintained or increased the level of +1 frame translation. In contrast, mutating G6568 specifically inhibited +1 frame translation.

We wished to determine if this trend is specific to IAPV or whether the other Type II IGR IRESs like KBV, SINV-1 and ABPV, which can direct +1 frame translation (4), also exhibited similar nucleotide dependencies. As predicted, disruption of the PKI base pairing within each Type II IGR IRES eliminated both 0 and +1 frame translation (Supplementary Figure S3B and C), indicating that +1 frame translation is IGR IRES-dependent. Furthermore, we deleted unpaired nucleotides within the KBV, SINV-1 and ABPV IGR IRESs at equivalent positions to A6554 and U6569 of the IAPV IGR IRES (Supplementary Figure S3A). Deletion of C6566 or C6581 within the KBV IGR IRES stimulated +1 frame translation by ∼2–4-fold (Supplementary Figure S3B and C). Similar deletions within the SINV-1 (ΔA4361 and Δ4376) and ABPV IGR IRESs (ΔA6475 and ΔU6490) resulted in either exclusive +1 frame translation or an increase in +1 to 0 frame translation ratio, but did not stimulate +1 frame translation (Supplementary Figure S3B and C). In general, as observed with the IAPV IGR IRES, the deletion of these nucleotides results in a preferential translation in the +1 frame translation over 0 frame translation, thus supporting the idea that a subset of Type II IGR IRESs uses similar determinants to direct reading frame selection.

#### Combinatorial mutations that direct exclusive 0 or +1 frame translation

Given that +1 frame translation is ∼20–25% of 0 frame translation, we propose that the wild-type IRES adopts distinct conformations in equilibrium prior to ribosome assembly and that specific mutations shift the equilibrium to favor an IRES conformation for 0 or +1 frame translation. To determine if a mutation that favors 0 or +1 frame translation is dominant over another, we created several double mutations. We combined G6618U mutation, which exclusively shows 0 frame translation, with either ΔA6554 or ΔU6569, both of which lead to +1 frame translation exclusively. The resultant double mutants conferred an intermediate level of 0 and +1 frame translation compared to that of the single mutation or deletion alone (Table 1, lines 7 and 8). Similarly, combining mutation U6562G, which increases +1 frame translation ∼4.5-fold, with mutation G6568C, which leads to a ∼95% decrease in +1 frame translation, resulted in intermediate levels of both 0 and +1 frame translation (∼80–90% translation in both frames compared to the wild-type IRES; Table 1, line 10). These results indicate that +1 frame translation directed by IRES mutants ΔU6569 or ΔA6554 depends on the adjacent base pairing between U6562 and G6618. Thus, one class of mutation is not dominant over another.

**Table 1.** Distinct class of IRES mutations that confer 0 or +1 frame translation

	0 frame	+1 frame
WT	100	100
G6618U	165 ± 20	8 ± 0
G6568C	142 ± 9	9 ± 5
ΔU6569	0 ± 0	365 ± 16
ΔA6554	3 ± 2	106 ± 19
U6562G	67 ± 6	419 ± 19
ΔU6569/G6618U	56 ± 8	38 ± 6
ΔA6554/G6618U	47 ± 7	21 ± 7
G6568C/G6618U	227 ± 18	2 ± 1
U6562G/G6568C	81 ± 9	90 ± 2
U6562G/ΔU6569	3 ± 1	530 ± 39
U6562G/ΔA6554	2 ± 1	278 ± 27

Quantitation of mutant IAPV IRES-mediated 0 and +1 frame translation in Sf21 extracts using bicistronic reporter constructs. The percent translation is normalized to the 0 and +1 frame translation (both as 100%) mediated by the wild-type IAPV IGR IRES. The results are from at least three independent experiments ± SD.

Finally, we asked whether mutations that increase 0 or +1 frame translation are additive. Combining mutations U6562G and ΔU6569 resulted in a slight increase in +1 frame translation compared to U6562G alone (Table 1, line 11). However, combinations of U6562G /ΔA6554 did not result in an additive effect on +1 frame translation compared to that of U6562G mutant IRES alone (Table 1, line 12) but did lead to complete loss of 0 frame translation. Similarly, combining mutations G6568C and G6618U, both of which decrease +1 frame translation, resulted in a minor increase in 0 frame translation (Table 1, line 9). These results suggest that these classes of mutations are at or close to maximal activity for +1 frame translation.

#### Nucleotide identity within the VLR affects IRES translation

SHAPE analyses showed differential reactivities within the nucleotides of the VLR upon ribosome binding (Figure 4). We asked whether mutations within the VLR affected IRES-driven translation. Altering AA6606-7 to UU or CC resulted in a general decrease in both 0 and +1 frame translation by ∼25–40% (Figure 5, gray box). Similarly, mutating AUAC6607-10 also decreased 0 frame translation by 15–34% without an effect on +1 frame translation (Figure 5, gray box). The overall decrease in translation of some mutations in the VLR is in agreement with previous results that the identities of specific nucleotides are important for IRES activity (24). It remains to be investigated how the VLR plays a role in IRES activity.

### IAPV IGR IRES-mediated 0 and +1 frame translation in cells

**Figure 6.**
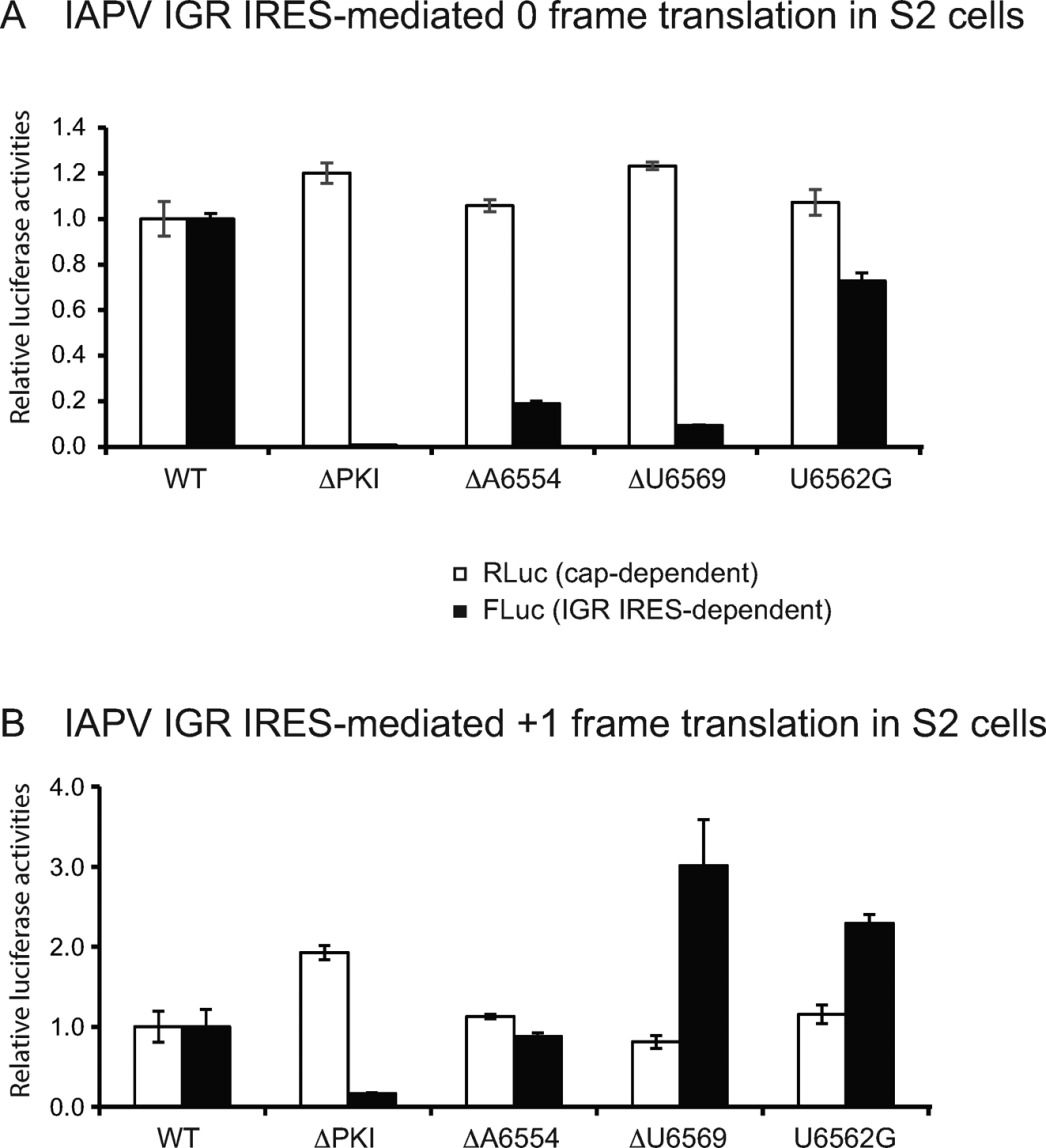
IAPV IGR IRES-mediated translation in *Drosophila* S2 cells. *In vitro* transcribed bicistronic reporter RNAs containing the wild-type or the indicated mutant IRESs that drive either (**A**) 0 frame or (**B**) +1 frame translation were transfected into *Drosophila* S2 cells. At 6 h after transfection, cells were harvested and lysed. Renilla (cap-dependent) and firefly (IRES-dependent) luciferase activities were measured and normalized to that of the wild-type IRES. The Fluc ORF was fused in the 0 or +1 frame to monitor IRES translation. Shown are averages from at least three independent experiments ± SD.

To determine if the observed uncoupling of 0 and +1 frame translation directed by mutant IRESs occurs *in vivo*, we transiently transfected *in vitro* transcribed capped bicistronic mRNAs containing either wild-type or mutant IRESs into *Drosophila* S2 cells and followed the translation of Renilla (cap-dependent) or Firefly (IRES-dependent) luciferase protein by measuring their enzymatic activities (Figure 6). We used a novel bicistronic reporter construct that increases the sensitivity of detection of IRES-mediated FLuc activity (20). As shown previously, the ΔPKI mutation, which disrupts PKI base pairing, significantly decreased FLuc activity in both 0 and +1 frames as compared to that of the wild-type IRES (Figure 6A and B) (20). We previously showed that the mutant IRES G6618U results in a decrease in +1 frame translation in *Drosophila* S2 cells (Wang,Q. and Jan,E., unpublished data). Transfection of reporter RNAs containing mutant IRESs ΔA6554, ΔU6569 and U6562G resulted in ratios of 0 to +1 frame translation that are similar to those observed in *in vitro* experiments (Figure 6). For example, ΔU6569 generated an ∼3-fold increase in +1 frame translation and an ∼90% decrease in 0 frame translation, which are similar to the effects observed *in vitro* (Figures 5 and 6). Consistent with these *in vitro* data, ΔA6554 maintained similar levels of +1 frame translation as the wild-type version and a decrease in 0 frame translation. U6562G showed an increase in +1 frame translation and ∼30% decrease in 0 frame translation (Supplementary Figures S1, S5 and S6). In summary, these results show that 0 and +1 frame translation mediated by the IRES can be uncoupled *in vitro* and in *Drosophila* S2 cells.

### SHAPE analysis of mutant IRESs

**Figure 7.**
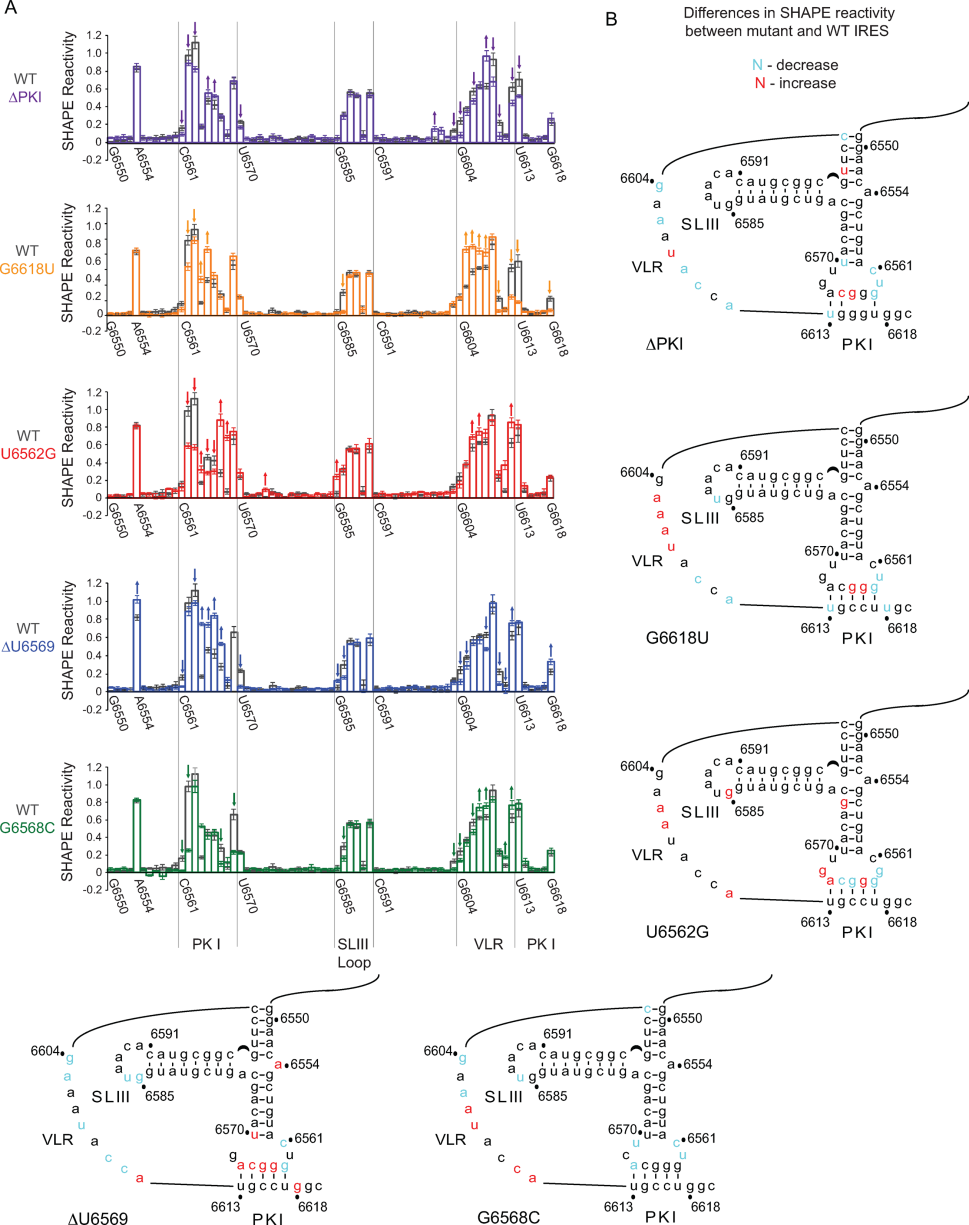
Structural analysis of mutant IAPV IGR IRESs by SHAPE. (**A**) Normalized NMIA reactivity profiles of the indicated mutant IRESs (colored bars) compared to the wild-type IRES (black bars). The nucleotide position/number is shown on the x-axis with major domains of the IRES denoted. The y-axis is normalized reactivity. Significant changes in NMIA reactivity in nucleotides between mutant and wild-type IRES are indicated with an arrow. (**B**) Secondary structures of mutant IRESs labeled with nucleotides that showed a significant increase (red) or decrease (blue) in NMIA reactivity within the mutant IRES compared to the wild-type IRES. Data represent the average of at least three independent experiments ± SD.

The mutational analyses reveal that IAPV IRES-mediated 0 and +1 frame translation can be uncoupled. We hypothesize that the IRES can assume a range of conformations, a subset of which favors one or the other specific reading frames. To address this possibility, we performed SHAPE analysis on mutant IRESs including G6618U and G6568C that lead to loss of +1 frame translation and U6562G and ΔU6569 that lead to an increase in or exclusive +1 frame translation. Supplementary Figure S4 shows the relative SHAPE reactivities of the mutant IRESs and in Figure 7, we superimpose the relative SHAPE reactivities of the mutant IRES onto that of the wild-type IRES. For comparison, SHAPE analysis of the mutant IRES ΔPKI, which disrupts PKI base pairing, resulted in changes in SHAPE reactivities within the PKI base pair region and VLR (Figure 7A, ΔPKI). For the G6618U mutant IRES, SHAPE reactivities were observed in the same regions in the wild-type IRES but to varying extents. Notably, we noted an increase in SHAPE reactivities at nucleotides 6605 to 6608 of the VLR and nucleotides 6564-5 of the PKI base pair in G6618U. Conversely, SHAPE reactivity decreased at nucleotides 6610 and 6612 of the VLR, at U6613 and UG6562-3 of the PKI base pair and at U6618 (Figure 7A). In contrast, the U6562G mutant IRES resulted in increased SHAPE reactivity at G6585 in the loop of SLIII, at G6564 and AG6567-8 of the loop of PKI and at G6574. The pattern of SHAPE reactivities within the VLR of the U6562G more closely resembled that of the wild-type IRES than G6618U, which may reflect the increased +1 frame translation observed with the U6562G mutant IRES (Supplementary Figure S4). SHAPE reactivities of the mutant IRESs ΔU6569 and G6568C differed from those of the wild-type IRES but, in general, the differences were subtle (Figure 7 and Supplementary Figure S4). For example, in ΔU6569, nucleotides GGCA6564-7 and U6570 of the PKI anticodon-like loop and the bulge A6554 showed increased SHAPE reactivities compared to the wild-type IRES (Figure 7). In summary, we observed changes in SHAPE reactivities between individual mutants and wild-type IRESs, suggesting that a subset of conformations, albeit subtle, is associated with 0 or +1 frame translation.

### SHAPE analysis of mutant IRES–ribosome complexes

**Figure 8.**
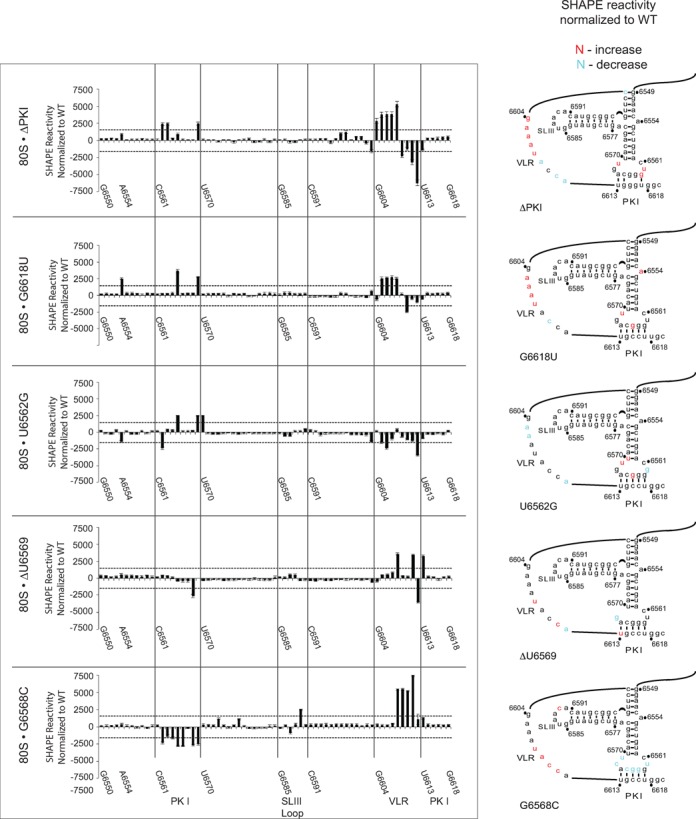
SHAPE analysis of mutant IAPV IGR IRESs in IRES–ribosome complexes. (**A**). Quantitation of the change in the NMIA reactivity of the indicated mutant IAPV IGR IRES in IRES-ribosome complexes compared to IRES in solution. The nucleotide position/number is shown on the x-axis with major domains of the IRES denoted. The difference in NMIA reactivity of each nucleotide within the PKI domain is shown. (**B**) Secondary structures of the PKI domain of mutant IAPV IGR IRESs are shown. Nucleotides that showed a significant increase (red) or decrease (blue) (greater than 1 SD) in NMIA reactivity are labeled. Data represent the average of at least three independent experiments ± SD.

We next interrogated the structure of the mutant IRESs bound to the ribosome by SHAPE analysis focusing on mutant IRESs that uncouple 0 and +1 frame translation. NMIA-mediated reactivities of nucleotides were compared between mutant IRES–ribosome and wild-type IRES–ribosome complexes. Nucleotides within a mutant IRES that showed an increase or decrease in SHAPE reactivity by more than two standard deviations compared to the wild-type IRES are shown in Figure 8. In general, the overall NMIA profiles of the mutant and wild-type IRESs bound to the ribosome are not significantly altered, indicating that the overall structures are similar in the IRES–ribosome complexes. However, subtle differences in SHAPE reactivities were observed within the PKI domain of each mutant IRES. For example, compared to the wild-type IRES bound ribosome, the ΔPKI mutant IRES displayed a significant increase in SHAPE reactivities at nucleotides 6604-8 within the VLR, at UG6562-3 and at U6569 and a decrease in reactivity at nucleotides 6609 and 6611-2 (Figure 8). The pattern of altered reactivities appears to be associated with an IRES that cannot position the ribosome correctly for translation (Figure 8). Interestingly, mutants G6618U and G6568C, which display exclusive IRES-mediated 0 frame translation, exhibited different NMIA-reactive profiles. G6618U showed a significant increase in reactivity at nucleotides 6605-8 within the VLR, at A6554, and at G6565 and U6569 within the PKI anticodon-like domain (Figure 8). In contrast, the G6568C mutant showed a general decrease in NMIA sensitivity at several nucleotides within the PKI base pairing domain (Figure 8), suggesting that the PKI base pairing domain is less flexible, which may contribute to IRES-mediated 0 frame translation. Though both direct exclusive 0 frame translation, the NMIA-reactive modification patterns between mutant IRESs G6618U and G6568C are different (Figure 8), which may reflect the specific type and location of the mutation within the PKI domain. Alternatively, the differences in 0 frame translation directed by G6618U and G6568C mutant IRESs may be associated with the distinct modification profiles (Figures 2D and 5). For the mutant IRESs U6562G and ΔU6569 that showed an increase or exclusive +1 frame translation, the SHAPE patterns are distinct (Figure 8). The interpretation of the SHAPE modifications of U6562G is complicated by the fact that this mutant IRES still directs significant 0 frame translation. For the ΔU6569, which displays a 3.5-fold increase in +1 frame translation, we observed specific changes in SHAPE reactivity at nucleotides G6568, U6613, and at a few nucleotides within the VLR. Cumulatively, the mutant IRESs bound to the ribosome displayed specific NMIA-reactive profiles that differ from those of the wild-type IRES.

### Ribosome positioning on IRESs by toeprinting analysis

**Figure 9.**
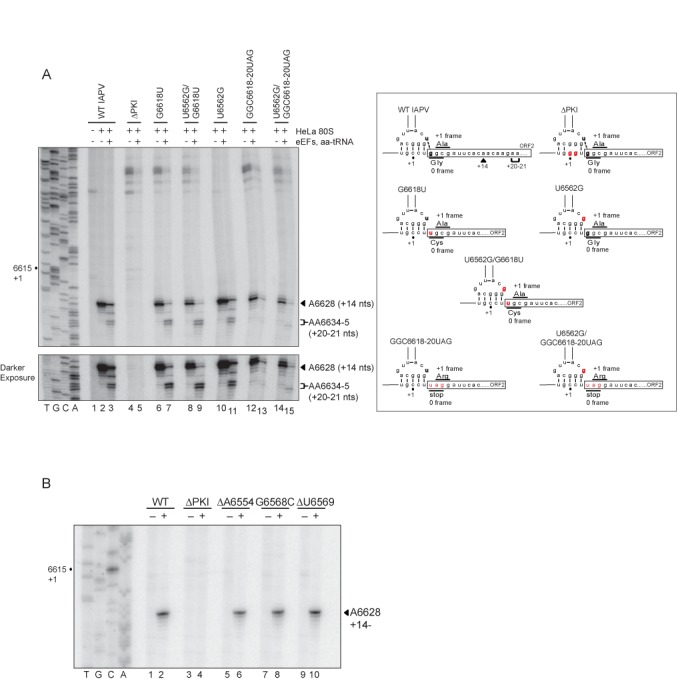
Toeprinting of IAPV IGR IRES/ribosome complexes. (**A**) (Left) Bicistronic RNAs containing wild-type or mutant IGR IRESs were incubated alone (lane 1) or with purified 80S subunits (lanes 2, 4, 6, 8, 10, 12, 14) or with purified 80S subunits, elongation factors eEF1A, eEF2 and bulk-aminoacyl-tRNAs (lanes 3, 5, 7, 9, 11, 13, 15). Cycloheximide is incubated with reactions to block elongation. Reactions were analyzed by primer extension analysis and separated by denaturing polyacrylamide gels. The gels were dried and exposed by autoradiography. The location of major toeprint at A6628 and the translocated toeprints are noted. Sequencing ladders for the wild-type (A) and mutant ΔPKI (B) IRESs are shown on the left, with their respective nucleotide numbers as indicated. (Right) A schematic of the wild-type and mutant IRESs and the location of the toeprints are shown. (**B**) Toeprints of purified ribosomes assembled on mutant IRESs ΔA6554, G6568C and ΔU6569. Representative gels are shown from at least three independent experiments.

The IAPV IRES directs ORFx translation by delivery of an Ala-tRNA to the ribosomal A site in the +1 frame (4). To determine whether 0 or +1 reading frame is selected upon IRES binding to the ribosome, we performed toeprinting analysis of ribosome/IRES complexes. Toeprinting analysis is a primer extension-based approach in which cDNA products can in principle be used to infer the nucleotides that occupy the ribosomal P and A sites and thus the starting translational reading frame of the ribosome on the IRES. We monitored the toeprints of purified salt-washed human ribosomes assembled on wild-type and mutant IAPV IGR IRESs. Assembly of ribosomes on the wild-type IGR IRES resulted in a strong toeprint at A6628 (Figure 9A, lane 2), which represents proper positioning of the ribosome on the IRES. Based on the original interpretation of these toeprints, toeprint A6628 is 14 nucleotides downstream of the CCU triplet given that the first C of the CCU is +1 and that the CCU triplet and the adjacent GGC glycine codon occupy the ribosomal P and A sites. However, in light of the recent cryo-EM studies showing that the PKI domain of the CrPV IGR IRES bound to the ribosome initially occupies the A site (15), the A6628 toeprint may be reinterpreted as 14 nucleotides downstream of the CCU triplet occupying the ribosomal A site. As expected, the ΔPKI mutant IRES eliminated the A6628 toeprint (Figure 9A, lane 4), in agreement with previous reports that the integrity of PKI is required for proper ribosome positioning on IGR IRESs (9,20). We first assessed the toeprints of assembled ribosomes on mutant IRESs G6618U, which abolishes +1 frame translation, and U6562G/G6618U and U6562G, which increase +1 frame translation. In each case, a strong toeprint at A6628 is reproducibly observed (Figure 9A, lanes 6, 8 and 10). Weaker toeprints are observed in some experiments at C6627 and A6629; however, the presence of these toeprints did not correlate with the ability of the IRES to direct 0 or +1 frame translation. Because +1 frame translation is weaker than 0 frame translation, it is possible that the toeprint for the +1 frame is masked. To circumvent this, we generated a mutant IRES where the starting 0 frame GGC glycine codon is replaced with a UAG stop codon, which should abolish both 0 and +1 frame translation (Figure 9A, right diagram). To restore +1 frame translation with this mutant IRES, we mutated U6562 to G, which mediates significant +1 frame translation (∼3.5-fold of wild-type IRES) (Figure 2D) (4). Both of these mutant IRESs produced a strong toeprint at A6628 similar to that observed with the wild-type IRES (Figure 9A, lanes 12 and 14). These results indicate that the position of the ribosome on the mutant IRESs is not altered and suggest that the reading frame is not differentially selected when ribosomes assemble on the IRES.

To confirm that the ribosome/IRES complexes are functional, we added purified yeast elongation factors, eIF1A and eEF2, and bulk aminoacyl-tRNAs to the reaction and monitored translocation by toeprinting (16). Addition of the factors to reactions containing the ribosome/wild-type IRES complex in the presence of cycloheximide produced two new toeprints at A6631 and AA6634-5 (Figure 7A, lane 3). The AA6634-5 toeprint is +6–7 nucleotides downstream of A6628 indicating that the ribosome has translocated by two codons, in agreement with previous reports showing that the ribosome can translocate two cycles of elongation in the presence of cycloheximide (8,16). The toeprint at A6631 is likely representative of an intermediate translocation event (16). Upon initiating translocation with ribosome/mutant IRES complexes, +6–7 toeprints were also observed at A6631 and AA6634-5 for mutants G6618U, U6562G and U6562G/G6618U, indicative of translocation events similar to that observed with the ribosome/wild-type IRES complex (Figure 9A, lanes 7, 9 and 11). As expected, the translocated toeprints were not observed with the mutant IRES containing the stop codon in the 0 frame (GGC6618–20UAG) (Figure 9A, lane 13), which is consistent with the model that the presence of the stop codon disrupts the adjacent U6562/G6618 base pairing. In contrast, the mutant IRES (U6562G/GGC6618–20UAG) that restores +1 frame translation displayed a weak yet reproducible toeprint at A6635 (Figure 9A, lane 15). The toeprint at A6635, which is +7 nucleotides downstream of A6628, represents ribosomes that have translocated through two cycles of elongation in the +1 frame. These results demonstrate that the toeprinting technique is sensitive enough to detect differences in 0 and +1 reading frames and that +1 frame translation mediated by the IAPV IRES can be reconstituted using minimal factors.

To further examine the reading frame selection by the IAPV IRES, we analyzed toeprints of ribosome assembled on mutants ΔA6554, G6568C and ΔU6569 that specifically uncouple 0 and +1 frame translation. Ribosomes assembled on these IRESs produced a strong toeprint at A6628 similar to that observed with the wild-type IRES (Figure 9B, lanes 6, 8 and 10). These results further support the conclusion that the +1 reading frame is not selected upon assembly of ribosomes on the IAPV IGR IRES.

## DISCUSSION

Recoding mechanisms expand the coding capacity of viral genomes and some cellular mRNAs. Most of these mechanisms involve RNA structures that interact with elongating ribosomes to affect the reading frame. For example, −1 and +1 programmed frameshifting utilizes pseudoknots, stem-loops and modified tRNAs that interact with the ribosome to shift the reading frame (2,25). Unlike mechanisms that recode during translational elongation, the IAPV IGR IRES is unique in that it directly recruits the ribosome and initiates translation in either the 0 or +1 reading frame, thus providing a simple yet elegant model for understanding how an RNA structure manipulates the ribosome and sets the reading frame. In this study, we provide extensive mutagenesis and biochemical analysis to investigate a new paradigm for recoding where the tRNA anticodon-like PKI domain of the IAPV IGR IRES adopts structural conformations that mediate reading frame selection.

Our previous findings demonstrated that the U6562/G6618 base pair adjacent to the anticodon-like PKI domain directs IAPV IRES-mediated +1 frame translation (4). These findings suggested a model where base pairing of the PKI domain measures out the reading frame similar to a ‘yardstick model’ to initiate in either the 0 or +1 frame depending on the frequency of base pairing of U6562/G6618. In this study, we demonstrate that in specific contexts mutating U6562 to other bases can still support +1 frame translation (Figure 2) (4), indicating that the adjacent base pairing is not absolutely necessary for +1 frame translation. At first glance, this appears to be contradictory to our previous findings. However, we argue that mutating G6618 does not alter the native conformation of the PKI anticodon-like loop, whereas mutating U6562 to the other bases likely alters the conformation of the PKI anticodon-like loop and thus affects reading frame selection. For instance, mutating U6562 to G resulted in a dramatic increase in +1 frame translation and induces subtle conformational changes of the mutant IRES in solution (Figures 2 and 7). In contrast, the wild-type IAPV IRES, which contains a U at nucleotide position 6562, adopts a native conformation that still requires base pairing at U6562/G6618 to direct +1 frame translation. Alternatively, we cannot formally rule out the possibility that a non-Watson–Crick interaction between nucleotides 6562 and 6618 contributes to +1 frame translation.

Our results revealed that novel mutations within single-stranded regions of the IGR IRES can uncouple IRES-mediated 0 and +1 frame translation (Figure 5). One class of mutants including G6618U, G6618C and G6568 to other bases results in exclusive 0 frame translation while the other class including ΔA6554 and ΔU6569 leads primarily to +1 frame translation (Figures 2 and 5). We predicted that each class of mutant IRES would have similar structural conformations to direct 0 or +1 frame translation. However, the differences among the SHAPE reactivity profiles of the wild-type and mutant IRESs are subtle, in solution and in IRES–ribosome complexes (Figures 7 and 8). This result suggests that the PKI domain is flexible and can adopt several if not multiple conformations, even when the ribosome is assembled on the IRES. One interpretation is that the distinct conformations and thus flexibility of the PKI domain are important for reading frame selection. Within each class of mutant IRES, the set of preferred conformations may represent intermediates that lead to selective translation in either the 0 or the +1 frame. For instance, mutation at ΔA6554, which is located at a triple helical junction, may promote an IRES conformation that differs from those of the distal mutation at ΔU6569 found within the PKI anticodon-like loop. However, the end outcome is the same—exclusive translation in the +1 frame. Conformational flexibility has been observed in suppressor tRNAs that mediate miscoding and stop codon read-through (26). The most notable examples are the Hirsh suppressor tRNA, which contains a G24A mutation in the D-Stem of tRNA^Trp^, and a suppressor tRNA containing a A9C mutation, both of which lead to stop codon read-through. Crystal structures of these suppressor tRNA bound to the ribosome revealed that each tRNA adopts a unique conformation to mediate miscoding: the A9C mutant tRNA increases the flexibility of the tRNA, whereas the G24A leads to an extra hydrogen bond that stabilizes the distortion of tRNA, which is required for decoding in the ribosomal A site (26). Similarly, it is possible that the mutations in the IAPV PKI tRNA-like domain adopt a subset of conformations to mediate +1 frame translation. It has been shown that a P/E hybrid tRNA may destabilize anticodon:codon interactions in the P site (27). Because crystallographic and ribosome docking studies have suggested that the IGR IRES may resemble a P/E tRNA hybrid-like state and thereby prime the ribosome for translation (13,14), it will be important to determine whether the dynamic nature of the IAPV IGR IRES PKI domain contributes to reading frame selection.

We considered other models such as P-site slippage or realignment of the PKI base pairing to explain the alternative reading frame selection by the IAPV IGR IRES. First, mutation of U6613 to A or U6617 to the other bases and thereby abolishing one base pair within the PKI domain moderately inhibits +1 frame translation, thus demonstrating that the integrity of the full five base pairs of the PKI domain is required for +1 frame translation (Figure 5). Furthermore, P-site slippage is not likely given that five base pairs within the PKI domain would have to slip to allow realignment of the reading frame. If the PKI domain slipped by +1, only two Watson–Crick base pairs would occur. Second, toeprinting analysis does not show a shift in the reading frame of ribosomes in complex with wild-type or mutant IRESs, which suggests that the reading frame has not been selected at this step (Figure 9). This conclusion is based on the sensitivity of the toeprinting approach to detect a change in reading frames, especially with the fact that +1 frame translation is only ∼20% of 0 frame translation. However, reconstitution of translation on mutant IRESs that only support +1 frame translation showed a shifted toeprint (+7) consistent with the ribosome translocating in the +1 reading frame (Figure 9). This result argues that the toeprinting assay is sufficiently sensitive to detect changes in reading frames. This result is also supported by other reports showing that the toeprinting technique can detect changes in reading frames of ribosomes complexed with tRNAs with expanded anticodon loops (28,29).

The PKI domain of the Type I CrPV IGR IRES structurally mimics a tRNA anticodon:codon interaction (13). The recent cryo-EM structure shows the PKI domain occupies the ribosomal A site prior to translocating to the P site (15). Here, the PKI domain of the IGR IRES docks precisely in the ribosomal P site and, in turn, selects the reading frame such that initiation occurs using a non-AUG codon in the A site. The PKI domain is thought to be dynamic and undergoes subtle structural changes upon binding to the ribosome, a property that may be intrinsic to IRES function (9,10,13,24). Similarly, we show that the PKI domain of the IAPV IGR IRES displays altered SHAPE reactivity profiles upon binding to the ribosome, suggesting that these conformational changes may be associated with not only IRES function but also reading frame selection (Figure 4B). The Type II IGR IRESs that include the IAPV are thought to be structurally similar to the Type I (30–32). Indeed, the PKI domains of Type I and II IGR IRESs are functionally interchangeable (33,34). However, notable differences between the Type I and II IGR IRESs, such as the presence of the SLIII and a longer anticodon-like loop within the Type II IGR IRES, may account for the ability of the IAPV IGR IRES to initiate translation in 0 and +1 frames.

We propose a new model whereby the IRES adopts distinct conformations in the ribosomal P site that direct 0 or +1 frame translation by occluding delivery of the 0 frame aa-tRNA to the A site and thereby allowing the delivery of the +1 frame aminoacyl-tRNA. The conformers of the IRES are in equilibrium such that the conformational subset that directs +1 frame translation is 20% of the conformation that directs 0 frame translation. Mutations at key locations of the PKI domain may shift the equilibrium to allow delivery of only the 0 or +1 frame aminoacyl-tRNA to the ribosomal A site. Given the recent report that the PKI domain may first occupy the ribosomal A site in IRES–ribosome complexes (15), it is possible that the IRES selects the reading frame from the ribosomal A site. However, from the toeprinting analysis, we propose that the reading frame has not been selected upon ribosome assembly on the IRES. Instead, the reading frame is selected after ribosome assembly when the PKI domain moves to and occupies the P site to drive translation by delivery of the incoming aminoacyl-tRNA by eEF1A, thus locking the ribosome to initiate in the 0 or +1 frame. We also propose that the IRES adopts distinct conformations in solution prior to ribosome recruitment, which may prime the ribosome to initiate translation in either 0 or +1 frame. In support of this, SHAPE analysis reveals distinct yet subtle conformational changes within the single-stranded regions of the IRES that may be associated with 0 or +1 frame translation (Figure 7). Previous reports have shown that the IGR IRES adopts a pre-formed structure to recruit the ribosome (11). Upon ribosome binding, the IRES undergoes further subtle conformational changes that mediate 0 or +1 frame translation (Figure 8). Further interrogation of the structures of the IAPV IGR IRES at steps after ribosome assembly (i.e. delivery of aminoacyl-tRNA and translocation) should provide further insights into reading frame selection by the viral IRES.

The IAPV IGR IRES directs +1 frame ORFx translation at ∼20% of 0 frame translation *in vitro* and ∼10–20% in virus-infected cells (Wang,Q., unpublished data). Although the function of ORFx is not known, ORFx is expressed in virus-infected honey bees, suggesting that ORFx facilitates virus infection (4). As with most programmed recoding mechanisms in viral genomes, the exact ratio of frameshifted versus in-frame viral protein expression is tightly regulated. Alterations by mutations or other means that change the ratio can affect virus infection. Similarly, the ratio and timing of +1 and 0 frame translation mediated by the IAPV IGR IRES is likely important for virus infection. It will be important to determine whether *trans*-acting factors or specific pathways can modulate IAPV IGR IRES-mediated 0 and +1 frame translation during virus infection, possibly by affecting the conformation of the IRES. Other factors such as the P site environment can also play a role in the ribosomal ‘grip’ on the P site tRNA and in maintaining the translational reading frame (35–37). Additional studies on the specific conformations and interactions of the tRNA-like domain of the IAPV IGR IRES with the ribosome will shed light on determinants that direct reading frame selection.

Recoding in the +1 frame has been found in a number of systems. One example that draws the closest comparison to the IAPV IGR IRES mechanism is the Hepatitis C virus (HCV) IRES. Several reports have shown that an overlapping +1 frame ORF called F protein (also called ARF or core +1) just downstream of the HCV IRES is produced in infected HCV patients (39). However, the mechanism has been controversial as it has been shown that F may be translated through a +1 frameshifting event or by the HCV IRES (38–40). The function of F is not known and F is not necessary for infection in tissue culture cells or in a mouse model (39,41,42). In another example, a subset of mutant tRNAs that contains mutations in the anticodon stem or elbow can suppress +1 frameshift mutations (36), suggesting that the tRNA interacts with the ribosome to influence reading frame selection (36). For instance, ribosomal protein rpL5 and helix 69 of the rRNA are located close to the elbow and upper anticodon stem of the tRNA, respectively. Given that the dicistrovirus IGR IRESs contain domains that mimic a tRNA and occupy the tRNA sites of the ribosome, the IAPV IGR IRES may interact with the ribosome in a manner similar to that of some +1 frameshift suppressor tRNAs to affect reading frame selection (43). In general, most frameshift suppressor tRNA mechanisms involve slippage of a near cognate tRNA in the ribosomal P site thus shifting the reading frame by +1 (37,44). However, +1 frameshifting may occur in the absence of slippage such as the event described in the Ty3 transposon in yeast (45,46). The Ty3 +1 frameshifting event occurs during translational elongation, requires near-cognate base pairing between the anticodon and codon in the P site and involves slow recognition of the A-site cognate codon (45,46). In contrast to these +1 recoding mechanisms described, the IAPV IGR IRES, although it occupies the ribosomal P site, does not involve slippage or a slowly recognized codon in the A site. Rather, the IAPV IGR IRES directs +1 frame selection at the step of initiation, albeit the ribosome is initiating translation in the elongation phase, and adopts a structural conformation occupying the ribosomal P site to occlude the delivery of the 0 frame aminoacyl-tRNA to the A site. Thus, the IAPV IGR IRES mechanism represents an unprecedented mechanism to alter reading frame selection and may be a paradigm for understanding properties of some programmed recoding events.

## SUPPLEMENTARY DATA

Supplementary Data are available at NAR Online.

SUPPLEMENTARY DATA
