# Supplementary material for: Structural determinants of an internal ribosome entry site that direct translational reading frame selection
Source: Nucleic Acids Res. 2014 Jul 18;42(14):9366–82. doi: 10.1093/nar/gku622 (PMC4132737; doi:10.1093/nar/gku622)
Supplement: SUPPLEMENTARY DATA [file supp_42_14_9366__index.html]

Structural determinants of an internal ribosome entry site that direct translational reading frame selection — Structural determinants of an internal ribosome entry site that direct translational reading frame selection — SUPPLEMENTARY DATA 

# Structural determinants of an internal ribosome entry site that direct translational reading frame selection

## SUPPLEMENTARY DATA

**Files in this Data Supplement:**

- SUPPLEMENTARY DATA
