# Supplementary material for: Structural determinants of an internal ribosome entry site that direct translational reading frame selection
Source: Nucleic Acids Res. 2014 Jul 18;42(14):9366–82. doi: 10.1093/nar/gku622 (PMC4132737; doi:10.1093/nar/gku622)
Supplement: SUPPLEMENTARY DATA [file supp_gku622_Ren_Supplemental_REVISED_July_10_2014.pdf]

Expanded View

Supplemental Figure Legends

Figure S1 Mutational analysis of U6562 and G6618. (A) PKI domain of the IAPV IGR IRES. The CC6615-6GG mutations that disrupt PKI basepairing and the compensatory mutations that restore basepairing are shown. (B and C) Quantitation of the radiolabeled +1 frame ORFx-Fluc (white bars) and 0 frame sORF2 (black bars) proteins normalized to RLuc. The ratios are normalized to the 0 and +1 frame translation of the bicistronic construct containing the wild-type IAPV IGR IRES given as 1.0. The data are plotted by grouping nucleotide changes to either U6562 (B) or G6618 (C). Averages are shown from at least three independent experiments ± s.d.

Figure S2 SHAPE analysis of wild-type and mutant IAPV IGR IRESs. Representative sequencing gels, which focus on the PKI domain of the IAPV IGR IRES, are shown. Sequencing from reactions containing dideoxy nucleotides are shown in the left lanes with their respective nucleotides. The sequencing gel was dried and analyzed by phosphorimager analysis.

Figure S3 Mutations within a subset Type II IGR IRESs that direct 0 or 1 frame translation. A) Secondary structures of the PKI domain of Type II IGR IRESs, IAPV, KBV, ABPV and SINV-1 IGR IRES. B) A representative gel of IRES-mediated 0 and +1 frame translation. Bicistronic reporter constructs were incubated in Sf21 extracts at 30 °C for 120 min in the presence of [^{35}S]-

methionine. C) Quantitation of the radiolabeled +1 frame ORFx-Fluc (white bars) and 0 frame sORF2 (black bars) proteins normalized to RLuc. The ratios are normalized to the 0 and +1 frame translation of the wild-type IGR IRES given as 1.0. Averages are shown from at least three independent experiments ± s.d.

Figure S4 SHAPE analysis of mutant IAPV IGR IRESs. SHAPE data overlayed on the secondary structure of PKI domain of the wild-type and mutant IAPV IGR IRESs. The colour coding indicates the relative SHAPE reactivities of individual nucleotides. Nucleotides are coloured in black, green, orange, and red from unreactive (<0.2) and limited reactivity (0.2-0.5) to moderately (0.5-0.8) and highly reactive (>0.8). SHAPE results are from at least three independent experiments ± s.d.

Ren_Fig S1

A

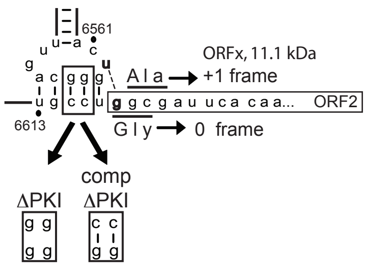

B

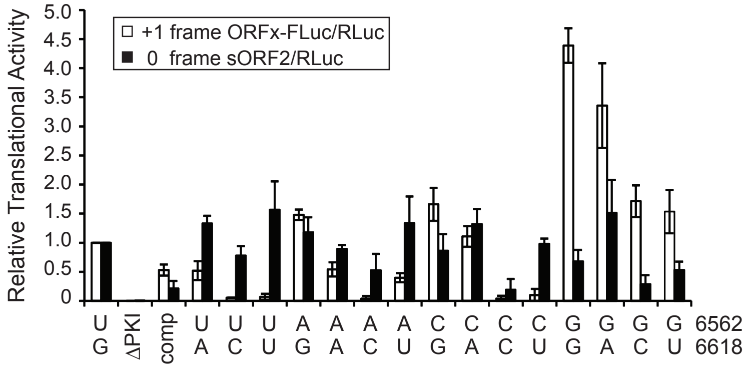

C

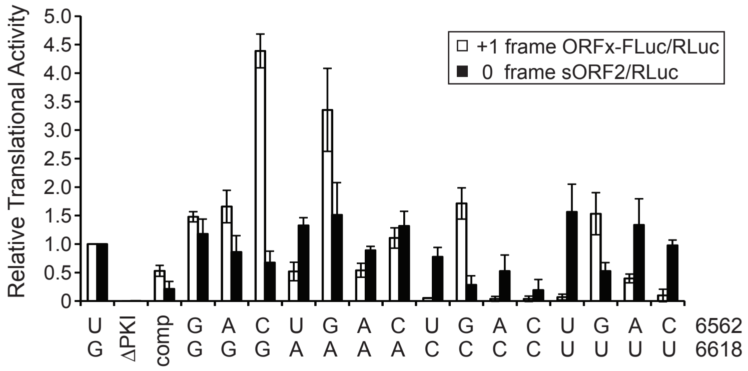

Ren_Fig. S2

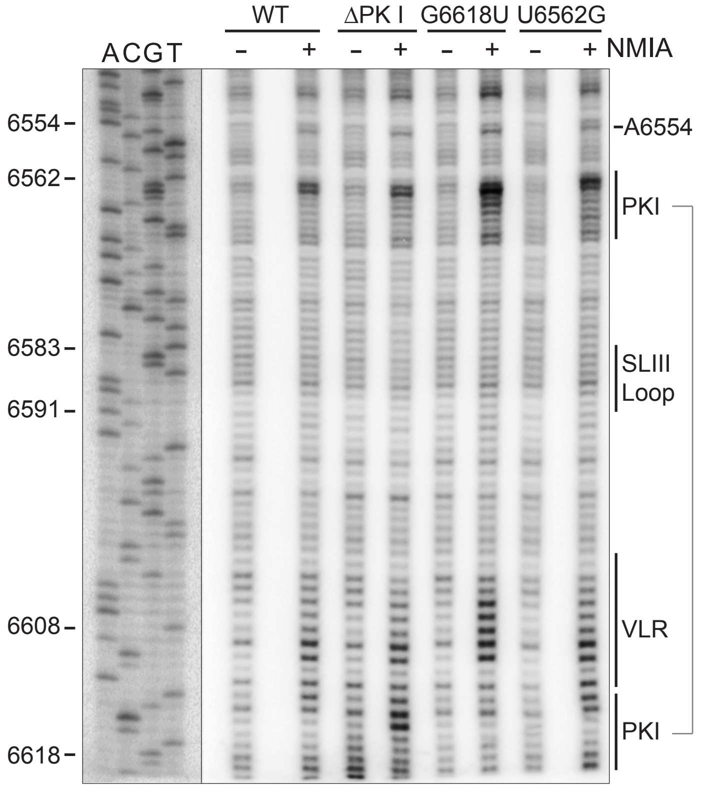
WT
ΔPK I
G6618U
U6562G
- + - + - + - + NMIA
ACGT
6554
6562
6583
6591
6608
6618
A6554
PKI
SLIII Loop
VLR
PKI

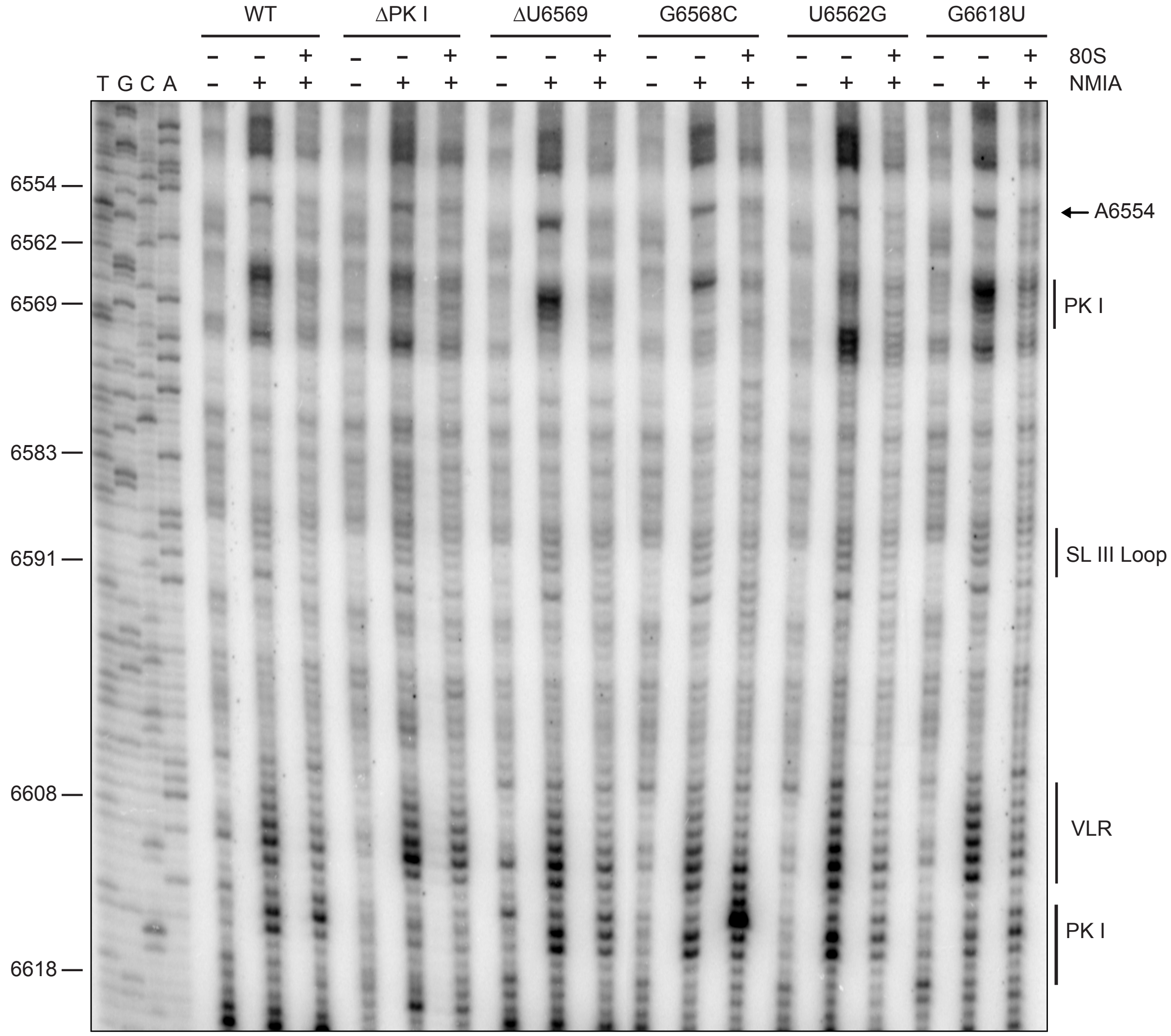
WT
ΔPK I
ΔU6569
G6568C
U6562G
G6618U
80S
NMIA
T G C A
6554
6562
6569
6583
6591
6608
6618
A6554
PK I
SL III Loop
VLR
PK I

Ren_Fig.S3

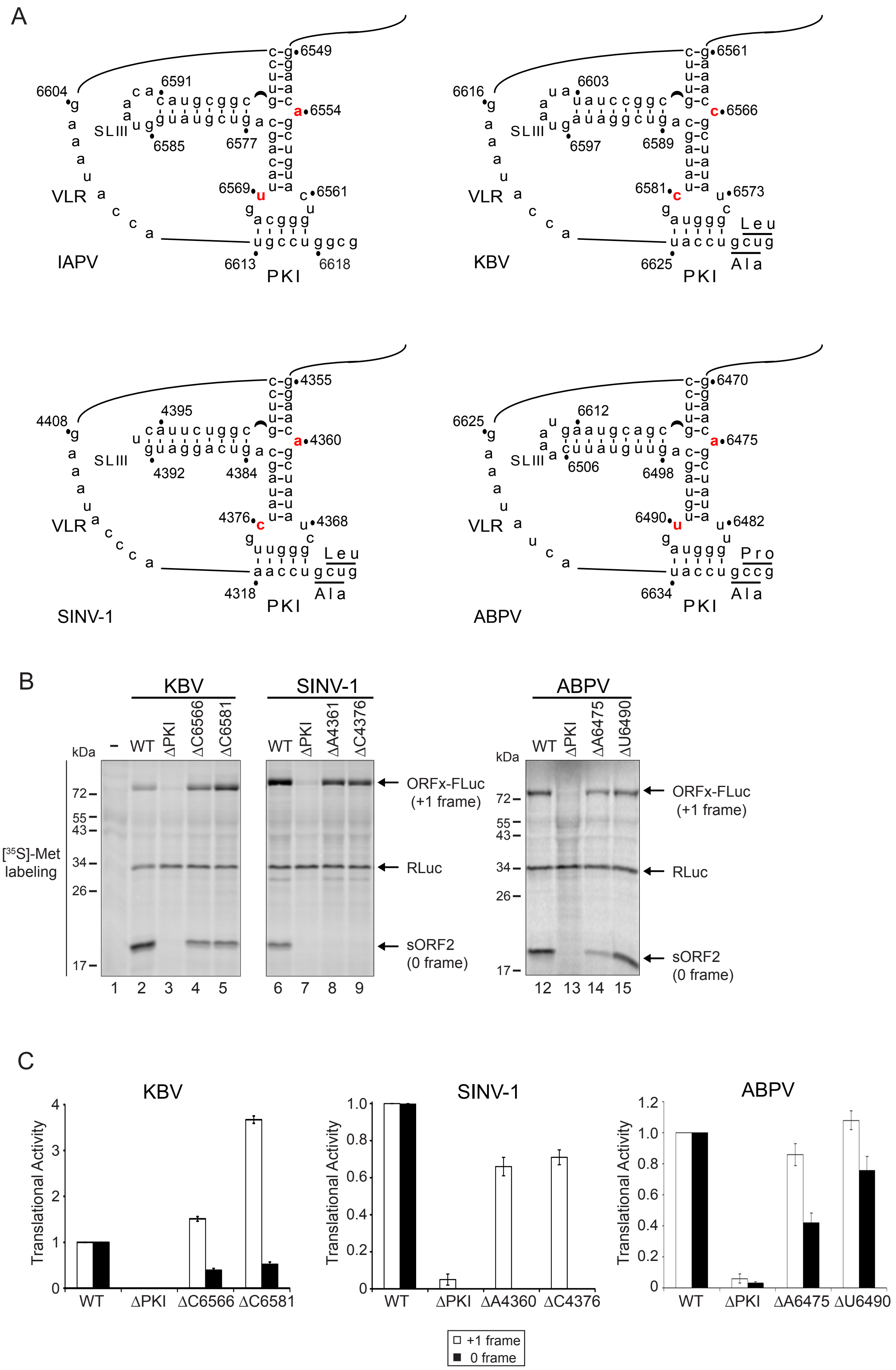

A
IAPV
KBV
SINV-1
ABPV
SLIII
VLR
PKI
Leu
Ala
Pro
B
KBV
SINV-1
ABPV
WT
ΔPKI
ΔC6566
ΔC6581
ΔA4361
ΔC4376
ΔA6475
ΔU6490
kDa
[35S]-Met labeling
ORFx-FLuc (+1 frame)
RLuc
sORF2 (0 frame)
C
KBV
SINV-1
ABPV
Translational Activity
WT
ΔPKI
ΔC6566
ΔC6581
ΔA4360
ΔC4376
ΔA6475
ΔU6490
+1 frame
0 frame

Ren_Fig. S4

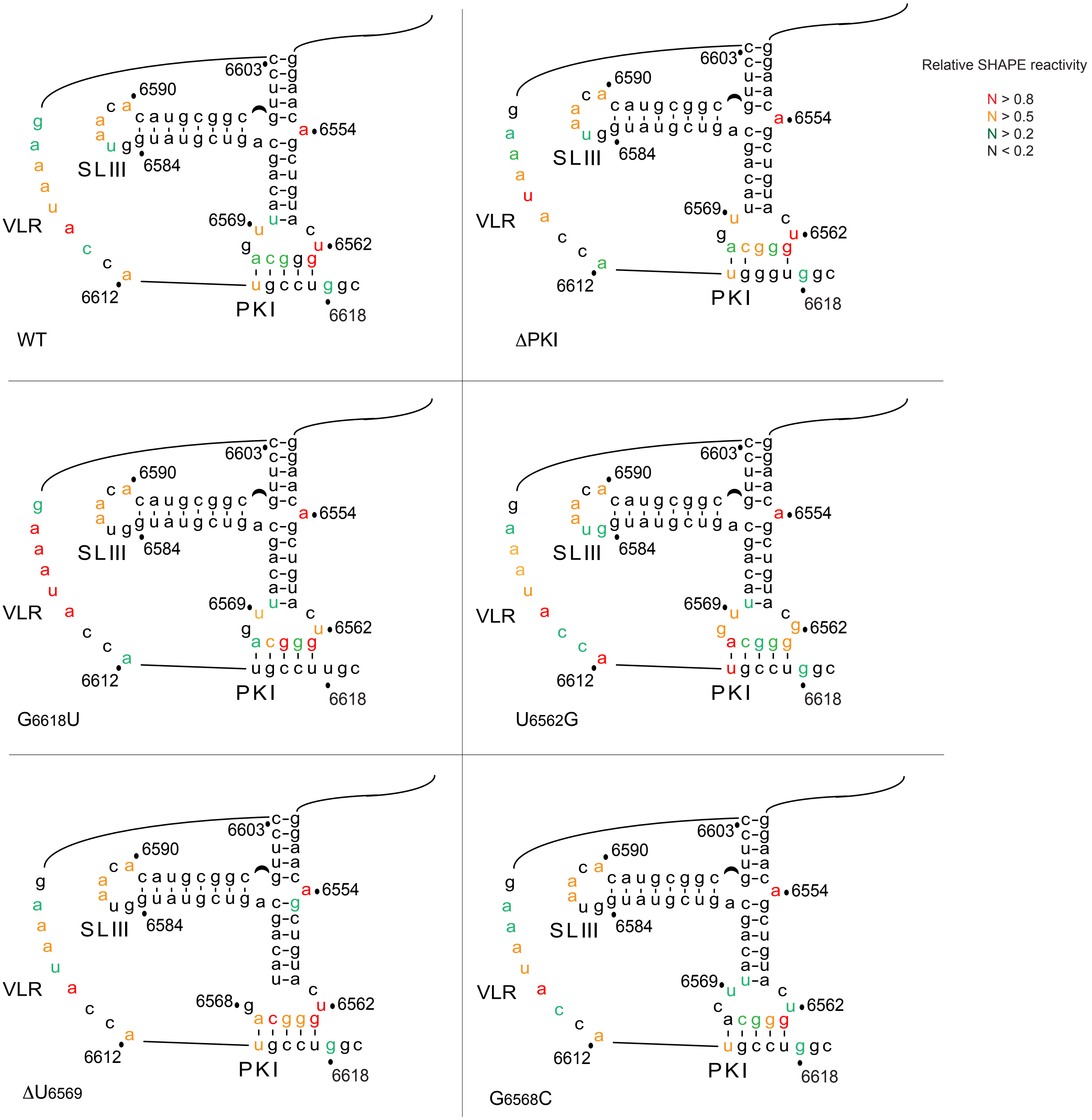
Relative SHAPE reactivity
N > 0.8
N > 0.5
N > 0.2
N < 0.2
SLIII
VLR
PKI
6603
6590
6584
6554
6569
6562
6612
6618
6568
WT
ΔPKI
G6618U
U6562G
ΔU6569
G6568C
